# Discontinuing renin-angiotensin system inhibitors after incident hyperkalemia and clinical outcomes: target trial emulation

**DOI:** 10.1038/s41440-025-02218-8

**Published:** 2025-05-14

**Authors:** Nobuhiro Hashimoto, Yusuke Sakaguchi, Koki Hattori, Yuki Kawano, Takayuki Kawaoka, Yohei Doi, Tatsufumi Oka, Yasuo Kusunoki, Satoko Yamamoto, Masafumi Yamato, Ryohei Yamamoto, Isao Matsui, Masayuki Mizui, Jun-Ya Kaimori, Yoshitaka Isaka

**Affiliations:** 1 Department of Kidney Disease and Hypertension, Osaka General Medical Center, Osaka, Japan; 2 Department of Nephrology, Osaka University Graduate School of Medicine, Suita, Japan; 3 Department of Nephrology, Yodogawa Christian Hospital, Osaka, Japan; 4 Department of Nephrology, Toyonaka Municipal Hospital, Toyonaka, Japan; 5 Department of Nephrology, Ikeda City Hospital, Ikeda, Japan; 6 Department of Nephrology, Sakai City Medical Center, Sakai, Japan; 7 Health and Counseling Center, Osaka University, Toyonaka, Japan

**Keywords:** Hyperkalemia, Chronic kidney disease, Discontinuing renin-angiotensin system inhibitors, Kidney outcome, Implemental hypertension

## Abstract

Although renin-angiotensin system inhibitors (RASi) are the mainstay in the management of heart failure with reduced ejection fraction, chronic kidney disease, and other cardiovascular conditions, they are often discontinued due to hyperkalemia. The prognostic impact of discontinuing RASi after developing hyperkalemia remains uncertain. Using a target trial framework based on the cloning, censoring, and weighting method, we compared discontinuing RASi after incident hyperkalemia with continuing RASi. We identified 2305 patients with an estimated glomerular filtration rate (eGFR) of ≥10 ml/min/1.73 m^2^ who developed hyperkalemia (serum potassium levels ≥5.5 mEq/L) while on RASi in the Osaka Consortium for Kidney Disease Research (OCKR) database. The primary outcome was a composite of initiation of kidney replacement therapy, a ≥50% decline in eGFR, or reaching eGFR <5 ml/min/1.73 m^2^. Secondary outcomes included all-cause death and severe hyperkalemia (serum potassium levels ≥6.5 mEq/L). The mean (standard deviation) age and eGFR were 68 (14) years and 29 (17) mL/min/1.73 m², respectively. After developing hyperkalemia, 346 (15%) discontinued RASi. Discontinuing RASi was associated with a 16% [95% confidence interval 2–33%] higher hazard of mortality than continuing RASi while the composite kidney outcome did not differ between groups (adjusted hazard ratio [HR] 1.01 [0.81–1.26]). Severe hyperkalemia occurred less frequently in those who discontinued RASi than those who continued RASi (adjusted HR 0.83 [0.69, 0.99]). RASi discontinuation after incident hyperkalemia was associated with higher mortality despite a lower risk of severe hyperkalemia. It was not related to kidney outcome. Appropriate clinical decision-making regarding RASi discontinuation may depend on the clinical context.

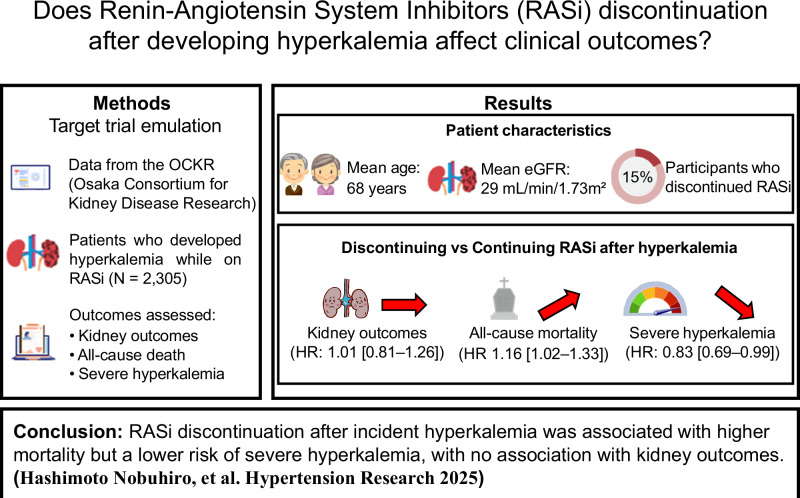

## Introduction

Over the last three decades, renin-angiotensin system inhibitors (RASi) have been the mainstay in the treatment strategy of heart failure with reduced ejection fraction (HFrEF), chronic kidney disease (CKD), and other cardiovascular conditions. Randomized controlled trials (RCTs) have shown the efficacy of RASi in improving outcomes and delaying disease progression in HFrEF, CKD, and other cardiovascular conditions [1–7]. Despite their established clinical benefits, however, RASi are often discontinued mainly due to hyperkalemia [8]. Although the latest Kidney Disease Improving Global Outcomes (KDIGO) guideline favors continuing RASi after developing hyperkalemia if it is controllable [9], there is a lack of clear evidence supporting the benefit of this strategy. The STOP-ACEi trial found that continuing RASi after an estimated glomerular filtration rate (eGFR) decreased to <30 ml/min/1.73 m^2^ did not significantly affect kidney outcomes [10], although this trial did not specifically address hyperkalemia-related RASi discontinuation. We have previously reported that restarting RASi after discontinuation was associated with better kidney outcomes and survival [11]. In this study, however, hyperkalemia-related RASi discontinuation was not the primary reason for RASi discontinuation since the proportion of patients who exhibited hyperkalemia at RASi discontinuation was quite low. More importantly, the notion that restarting RASi is beneficial does not have a direct influence on the clinical decision-making regarding whether to continue or discontinue RASi when hyperkalemia develops.

Several observational studies compared continuation versus discontinuation of RASi after the onset of hyperkalemia [12–15]. Unfortunately, these studies were limited due to selection bias arising from a landmark study design [16], in which a proportion of patients dropped out during the landmark period. To address such a methodological issue, a target trial emulation framework has been developed. Using this sophisticated approach, several studies have examined the impact of hyperkalemia-related RASi discontinuation [17, 18]. However, these studies included individuals with relatively preserved kidney function and did not assess kidney outcomes. Thus, it remains unclear whether RASi should be continued or not when hyperkalemia develops in those with advanced CKD.

Here, we utilized a target trial emulation framework based on the cloning, censoring, and weighting method to analyze the association between RASi discontinuation after developing hyperkalemia and clinical outcomes including kidney prognosis in a broader patient population.

Point of view

- Clinical relevance: Balancing hyperkalemia control and RASi benefits is crucial for optimal patient outcomes.
- Future direction: Further research is needed on novel potassium binders and individualized RAS inhibitor regimens.
- Consideration for the Asian population: Considering the region’s relatively high burden of kidney failure, tailored renoprotective strategies are essential.

## Methods

### Target trial emulation

Survival analysis based on observational data presents several challenges, including immortal time bias and depletion of susceptible bias [19, 20]. To address these biases, we employed the target trial emulation based on the cloning, censoring, and weighting method. In this methodology, all outcome events that occur during a grace period are included in survival analyses, preventing the drawback of a landmark analysis, which excludes events occurring during a landmark period [16]. The detailed design of a hypothetical randomized trial is shown in Supplementary Table 1.

The study protocol was approved by the Ethics Committees at each hospital. The study adhered to the principles of the Declaration of Helsinki. As the study design was retrospective, the need for informed consent was waived (approval number: 21513).

### Date sources

Data used in this study were collected from the Osaka Consortium for Kidney Disease Research (OCKR) database, a retrospective multicenter cohort study, including patients followed up by nephrologists at five hospitals in Osaka, Japan (Osaka General Medical Center, Osaka University Hospital, Ikeda City Hospital, Sakai City Medical Center, and Toyonaka Municipal Hospital) from January 2005 to December 2021. Demographic, laboratory, and prescription data were extracted from both outpatient and inpatient settings using an electronic data capture (EDC) system securely linked with electronic medical records within each hospital [11, 21–23].

### Target trial inclusion and exclusion criteria

The inclusion criteria were patients 1) aged 20 years or older, 2) who were prescribed RASi, 3) who developed hyperkalemia (serum potassium levels ≥5.5 mEq/L) for the first time since RASi were prescribed (index day), and 4) whose eGFR was ≥10 mL/min/1.73 m² at the index day.

Patients were excluded from the study if they had undergone kidney replacement therapy (KRT).

### Treatment assignment

To align with real-world clinical setting, we allowed 183 days of a grace period between the onset of hyperkalemia and RASi discontinuation. Patients who discontinued RASi during the grace period were treated as a “Discontinuation group”, while those who did not discontinue were treated as a “Continuation group”.

The primary analyses were performed in a per-protocol manner, as a substantial proportion of patients who discontinued RASi during the grace period restarted them thereafter (32%). We also performed an intention-to-treat analysis as a sensitivity analysis.

### Study outcomes and follow-up

The primary outcome was a composite kidney outcome consisting of KRT initiation, a ≥ 50% decline in eGFR from the index day, or kidney failure (eGFR <5 mL/min/1.73 m^2^). We ascertained KRT initiation using our record of incident dialysis patients in which all patients who initiated KRT in our hospitals were registered. Few patients initiated KRT outside our hospitals. Secondary outcomes included all-cause death and severe hyperkalemia (serum potassium levels ≥6.5 mEq/L). Deaths were extracted using the EDC system, which included in-hospital and out-of-hospital deaths.

Patients were followed up from the index day when hyperkalemia developed for the first time since RASi were initiated until the first outcome event, loss to follow-up, the end of the study period (31st December 2021), or up to five years after the index day.

### Baseline and time-varying covariates

Baseline covariates included age, sex, body mass index (BMI), and diabetes mellitus (DM), systolic blood pressure, diastolic blood pressure, laboratory measurements (hemoglobin, sodium, potassium, eGFR, calcium, phosphorus, urate, venous bicarbonate, urine protein-to-creatinine [Cre] ratio [UPCR]), and medications (angiotensin-converting enzyme inhibitors [ACEIs], angiotensin II receptor blockers [ARBs], mineralocorticoid receptor antagonists [MRAs], calcium channel blockers, beta-blockers, alfa-blockers, statins, sodium-glucose cotransporter 2 inhibitors, loop diuretics, thiazide diuretics, sodium bicarbonate, potassium binders, laxatives, and non-steroidal anti-inflammatory drugs). The laboratory measurements and medications were treated as time-varying covariates. DM was defined as baseline HbA1c ≥ 6.5% or use of glucose-lowering drugs at baseline. eGFR was calculated using a Japanese standard formula: 194 × serum creatinine^−1.094^ × age^−0.287^ (if female, ×0.739) [24].

### Statistical analysis

To emulate the target trial, we used the cloning, censoring, and weighting method (Supplementary Fig. 1). A specific dataset was created with two copies of each eligible individual, with one assigned to the Discontinuation group and the other to the Continuation group. Replicates were censored when they deviated from their assigned strategy during the 183-day grace period, which was monitored monthly. Replicates in the Discontinuation group were artificially censored at the end of the grace period if RASi were not discontinued. Replicates in the Continuation group were artificially censored on the day when RASi were discontinued during the grace period. A selection bias potentially introduced by artificial censoring was addressed using a time-varying inverse probability of censoring weights (IPCW). The IPCW were the reciprocal of the probability of being uncensored, estimated from a pooled logistic regression model including baseline and time-dependent covariates as well as time (linear and quadratic terms). The IPCW were stabilized by multiplying them by the probability of being uncensored based on pooled logistic regression models including baseline covariates only. The IPCW at each month were calculated by multiplying all IPCW from the index day up to a specific month. These weights create pseudo-populations where censoring was independent of measured confounders. IPCW were truncated at the 99th percentile to avoid an influence of significant outliers. A standardized mean difference (SMD) was calculated to evaluate between-group differences in covariates at the end of the grace period. The SMD of less than 0.1 indicates a negligible difference. Finally, we employed IPCW-weighted pooled logistic regression models to estimate the association between the treatment groups and outcomes. The models included an indicator for the treatment groups, time (linear and quadratic terms), and baseline covariates with a robust variance estimator. The pooled logistic regression is known to provide equivalent estimation for hazard ratios (HR) and 95% confidence intervals (CI) based on a Cox model [25, 26]. Predicted probabilities derived from the logistic models were utilized to estimate absolute risk difference [27].

To impute all baseline missing values, multiple imputation by chained equations (MICE) with fully conditional specification was utilized. The imputation model included all baseline covariates. As all covariates with missing values were continuous variables (BMI, hemoglobin, sodium, calcium, phosphorus, urate, bicarbonate, and UPCR), linear regression imputation was performed to create 20 imputed datasets. For each of these imputed datasets, 200 bootstrap samples were generated. The IPCW-weighted pooled logistic regression models were applied to each of the 20 × 200 datasets. The 95% CIs were based on the 2.5th and 97.5th percentiles of the pooled distribution of the bootstrap point estimates [28].

Since laboratory tests were performed every 1–3 months in general, we collected these data on a monthly basis, imputing missing data using the last observation carried forward method.

### Subgroup and sensitivity analyses

We performed subgroup analyses after dividing patients by age (≥70 vs. <70 years), sex, baseline eGFR (<30 vs. ≥30 mL/min/1.73 m^2^), UPCR (<1 vs. ≥1 g/gCre), and DM. The interaction term between the treatment groups and these potential effect modifiers was incorporated into the IPCW-weighted pooled logistic regression models.

We performed several sensitivity analyses to test the robustness and consistency of our results. Firstly, we shortened the grace period from 183 to 100 days. This would reduce the number of patients discontinuing RASi within the grace period, while potentially increasing the probability that RASi were discontinued due to hyperkalemia itself. Second, we repeated the same analysis in the intention-to-treat population. In the Discontinuation group, replicates were not censored if RASi were resumed after the grace period. In the Continuation group, replicates were not censored if RASi were discontinued after the grace period. Finally, we excluded bicarbonate from the covariates of all analyses due to a substantial number of missing data.

All statistical analyses were conducted using Stata/IC 18.0 software (Stata Corp, College Station, TX, USA). A *P*-value of less than 0.05 was considered statistically significant.

## Results

### Baseline characteristics

**Fig. 1:**
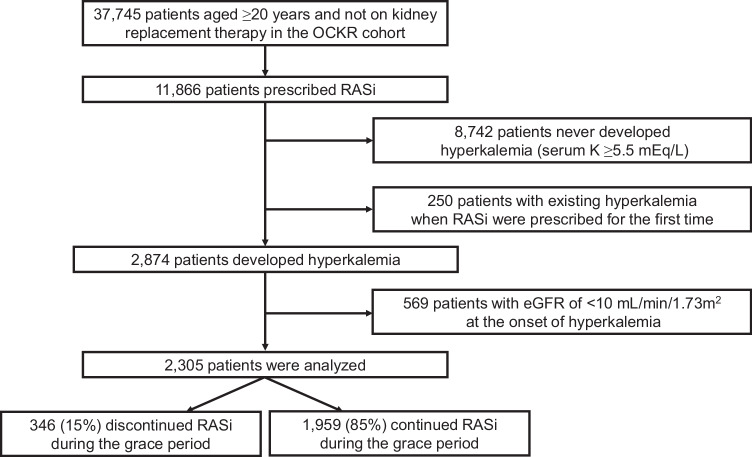
Flow diagram of the study participants. RASi renin-angiotensin system inhibitors, eGFR estimated glomerular filtration rate

Figure 1 shows the flowchart of this study. Of 11,866 patients who were prescribed RASi, 2305 (19%) developed hyperkalemia (serum potassium levels ≥5.5 mEq/L). The mean follow-up was 875 (standard deviation [SD], 653) days. At the onset of hyperkalemia (index day), the mean (SD) age and eGFR were 68 (14) years and 29 (17) mL/min/1.73 m², respectively. The median (interquartile range) UPCR was 1.2 (0.4, 2.9) g/gCre (Table 1).

**Table 1:** Demographic and clinical characteristics at the onset of hyperkalemia

Characteristics	The number (%) of missing data	Overall cohort (*n* = 2305)
*Demographics*		
Age (years)	0	68 (14)
Sex, male	0	1545 (67%)
Body mass index (kg/m^2^)	150 (7%)	22.7 (4.5)
Diabetes mellitus	0	967 (42%)
Systolic blood pressure (mmHg)	398 (17%)	137 (22)
Diastolic blood pressure (mmHg)	403 (17%)	76 (14)
*Laboratory values*		
Hemoglobin (g/dL)	3 (<1%)	11.2 (1.9)
Sodium (mEq/L)	8 (<1%)	139 (4)
Potassium (mEq/L)	0	5.8 (0.4)
eGFR (mL/min/1.73m^2^)	0	29 (17)
Calcium (mg/dL)	90 (4%)	8.8 (0.8)
Phosphorus (mg/dL)	261 (11%)	3.8 (0.8)
Urate (mg/dL)	37 (2%)	6.9 (1.8)
Bicarbonate (mEq/L)	1219 (53%)	23.5 (3.9)
UPCR (g/gCre)	700 (30%)	1.2 (0.4–2.9)
*Medications*		
ACEIs	0	716 (31%)
ARBs	0	1926 (84%)
MRAs	0	541 (23%)
Calcium channel blockers	0	1654 (72%)
Beta blockers	0	805 (35%)
Alfa blockers	0	262 (11%)
SGLT2 inhibitors	0	74 (3%)
Statins	0	663 (29%)
Loop diuretics	0	1056 (46%)
Thiazide diuretics	0	426 (18%)
Sodium bicarbonate	0	552 (24%)
Potassium binders	0	512 (22%)
Laxatives	0	881 (38%)
NSAIDs	0	37 (2%)

Data are presented as *n* (%), mean (standard deviation), or median (interquartile range)

*eGFR* estimated glomerular filtration rate, *UPCR* urinary protein-to-creatinine ratio, *ACEIs* angiotensin-converting enzyme inhibitors, *ARBs* angiotensin II receptor blockers, *MRAs* mineralocorticoid receptor antagonists, *SGLT2 inhibitors* sodium-glucose cotransporter 2 inhibitors, *NSAIDs* non-steroidal anti-inflammatory drugs

After developing hyperkalemia, 346 (15%) discontinued RASi within the 183-day grace period. The patient characteristics at the end of the grace period are shown in Supplementary Table 2. Before IPCW weighting, patients in the Discontinuation group were older and had lower BMI, hemoglobin, and UPCR. After weighting, all covariates, except for the use of thiazide diuretics, and potassium binding agents, were well balanced between groups.

### Discontinuation of RASi and clinical outcomes

After cloning, 621 replicates reached the composite kidney outcome, and 475 died during follow-up. There was no significant risk difference in the composite kidney outcome between the Discontinuation group and the Continuation group (adjusted hazard ratio (HR) 1.01; 95% confidence interval [CI] 0.81, 1.26) (Table 2). Similarly, the weighted 5-year absolute risk difference was not significant (0.6 [−3.7, 5.0]%) (Table 2). The results were consistent for each component of the composite kidney outcome (Table 2).

**Table 2:** Associations between RASi discontinuation after incident hyperkalemia and clinical outcomes

Outcomes	Hazard ratio (95% CI)	5-year absolute risk, % (95% CI)	5-year risk difference, % (95% CI)	5-year risk ratio (95% CI)
Primary outcome^a^				
Continuation group	Ref	20.4 (16.2, 24.1)	Ref	Ref
Discontinuation group	1.01 (0.81, 1.26)	20.7 (16.9, 24.9)	0.6 (−3.7, 5.0)	1.03 (0.81, 1.30)
KRT				
Continuation group	Ref	11.4 (8.6, 14.6)	Ref	Ref
Discontinuation group	0.93 (0.70, 1.24)	11.0 (8.3, 14.6)	−0.5 (−3.7, 2.6)	0.96 (0.71, 1.27)
eGFR ≥ 50% decline				
Continuation group	Ref	15.0 (12.0, 18.5)	Ref	Ref
Discontinuation group	1.03 (0.81, 1.33)	15.3 (11.4, 18.6)	0.1 (−4.1, 3.8)	1.00 (0.76, 1.31)
eGFR < 5 mL/min/1.73 m^**2**^				
Continuation group	Ref	6.4 (3.8, 9.4)	Ref	Ref
Discontinuation group	0.90 (0.60, 1.36)	5.8 (3.3, 8.3)	−0.5 (−3.1, 2.1)	0.90 (0.60, 1.42)
All-cause death				
Continuation group	Ref	39.1 (33.8, 43.7)	Ref	Ref
Discontinuation group	1.16 (1.02, 1.33)	45.6 (40.1, 50.7)	7.1 (1.3, 11.9)	1.18 (1.03, 1.33)
Severe hyperkalemia^b^				
Continuation group	Ref	30.1 (24.5, 36.0)	Ref	Ref
Discontinuation group	0.83 (0.69, 0.99)	24.1 (19.1, 28.9)	−6.2 (−12.5, −0.6)	0.80 (0.64, 0.98)

*CI* confidence interval, *KRT* kidney replacement therapy, *eGFR* estimated glomerular filtration rate, *RASi* renin-angiotensin system inhibitors

^a^A composite kidney outcome consisting of KRT initiation, a > 50% decline in eGFR from the index day, or kidney failure (eGFR <5 mL/min/1.73 m^2^)

^b^Severe hyperkalemia was defined as serum potassium levels of >6.5 mEq/L

The Discontinuation group showed a significantly higher hazard of all-cause mortality than the Continuation group (adjusted HR 1.16 [1.02, 1.33]). The weighted 5-year absolute risk difference was 7.1 [1.3, 11.9]% (Table 2). Severe hyperkalemia (serum potassium levels ≥6.5 mEq/L) occurred less frequently in the Discontinuation group than in the Continuation group (adjusted HR 0.83 [0.69, 0.99]) (Table 2).

**Fig. 2:**
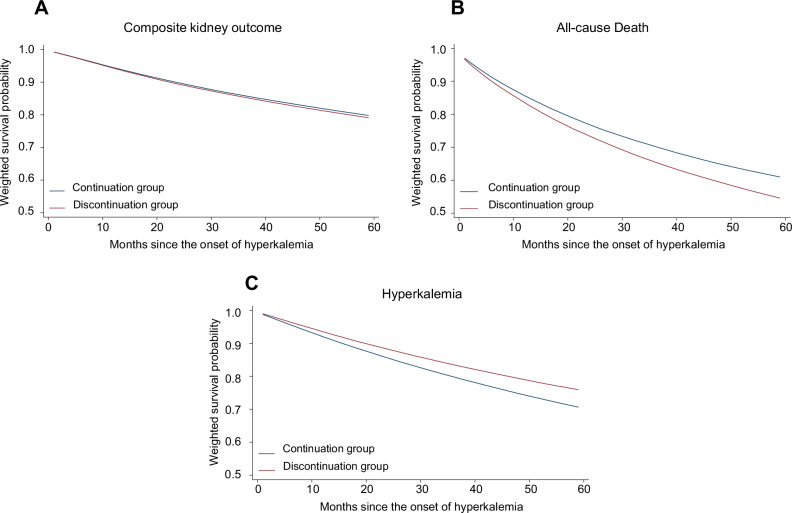
RASi discontinuation after incident hyperkalemia and clinical outcomes. IPCW-weighted survival probability curves show: **A** no significant difference in the composite kidney outcome between the Discontinuation and Continuation groups; **B** a significantly higher risk of all-cause death in the Discontinuation group compared to the Continuation group; and **C** a lower risk of severe hyperkalemia (serum potassium levels ≥6.5 mEq/L) in the Discontinuation group compared to the Continuation group. RASi renin-angiotensin system inhibitors, IPCW inverse probability of censoring weights, CI confidence intervals

The weighted survival probability curves for the composite kidney outcome, death, and severe hyperkalemia are shown in Fig. 2.

### Subgroup analyses

**Fig. 3:**
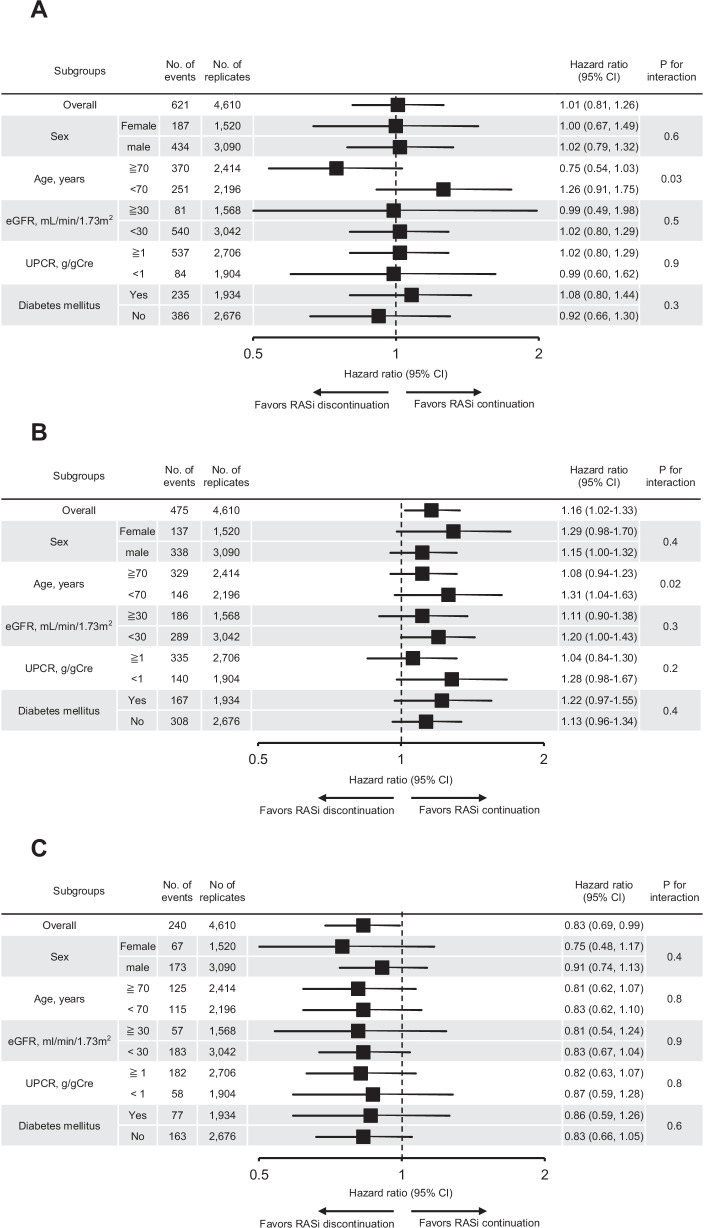
Subgroup analysis for clinical outcomes. **A** Composite Kidney Outcome No significant overall association is found between RASi discontinuation and the composite kidney outcome. However, a significant qualitative interaction by age is observed, with a trend toward an increased risk of the composite kidney outcome in patients under 70 years. Boxes and bars represent adjusted hazard ratios and 95% CI, respectively. **B** All-Cause Death No significant overall association is found between RASi discontinuation and all-cause death. However, a significant interaction by age is observed, with a trend toward an increased risk of all-cause death in patients under 70 years. Boxes and bars represent adjusted hazard ratios and 95% CI, respectively. **C** Severe Hyperkalemia The Discontinuation group consistently shows a lower hazard of severe hyperkalemia (serum potassium levels >6.5 mEq/L) across all prespecified subgroups. Boxes and bars represent adjusted hazard ratios and 95% CI, respectively. RASi renin-angiotensin system inhibitors, eGFR estimated glomerular filtration rate, UPCR urine protein-to-creatinine ratio, Cre creatinine, CI confidence interval

None of the prespecified covariates except for age modified the association between RASi discontinuation and the composite kidney outcome: the association was substantially stronger in patients under 70 years compared to those aged 70 years or older (Fig. 3A). A significant interaction was also observed for all-cause death, with a higher risk in patients under 70 years compared to those aged 70 years or older (Fig. 3B). No significant interaction was found between RASi discontinuation and severe hyperkalemia (Fig. 3C).

### Sensitivity analyses

Concerning the composite kidney outcome, the results were not substantially altered after 1) the grace period was shortened from 183 days to 100 days; 2) analyzing the follow-up data in an intention-to-treat manner instead of a per-protocol manner; and 3) omitting data on bicarbonate levels from the analysis (Supplementary Tables 3–5).

The Discontinuation group consistently showed a 14–16% higher hazard of all-cause death than in the Continuation group (Supplementary Tables 3–5).

## Discussion

Discontinuing RASi after developing hyperkalemia, frequently occurs in real-world clinical settings, has been a controversial issue [8]. Using the target trial emulation based on the cloning, censoring, and weighting method, we found that discontinuing RASi after the onset of hyperkalemia was not related to poorer kidney outcomes but was associated with higher mortality than continuing RASi. Conversely, RASi discontinuation was associated with a lower risk of severe hyperkalemia. Sensitivity and subgroup analyses yielded similar results. These findings provide a practical implication for the management of patients who develop hyperkalemia while on RASi. In particular, clinical decision-making may need to be individualized based on the trade-off between a lower risk of severe hyperkalemia and higher mortality associated with RASi discontinuation.

While RASi have been shown to improve kidney prognosis [4–6], we found a neutral association between RASi discontinuation after incident hyperkalemia and kidney outcome. Consistently, we have previously reported that restarting RASi after discontinuation was associated with better kidney outcomes in a whole cohort but not in those with a history of hyperkalemia [11]. Unlike our findings, several studies reported that hyperkalemia-related discontinuation of RASi was associated with a higher risk of KRT initiation [12, 15]. One of the drawbacks of their landmark analysis was selection bias as only patients who survived without maintenance dialysis therapy for more than 90 days after incident hyperkalemia were analyzed [16]. For example, those who continued RASi but eventually underwent dialysis within the landmark period due to hyperkalemia were excluded from their analysis. To overcome this methodological issue, we utilized the cloning, censoring, and weighting method in which all outcome events that occurred during the grace period were included in survival analyses. Another limitation of these studies was the lack of adjustment for several essential drugs for hyperkalemia (potassium binders, diuretics, and sodium bicarbonate), urinary protein, and serum potassium levels during follow-up, all of which were adjusted in our survival analyses [12, 15]. These differences might explain the discrepancies in the results between the two studies.

The neutral association between RASi discontinuation after incident hyperkalemia and kidney outcome may imply that hyperkalemia diminishes the kidney protective effect of RASi. Notably, hyperkalemia induces aldosterone secretion independently of the renin-angiotensin system [29, 30]. Thus, it can be speculated that RASi-related hyperkalemia may contribute to aldosterone breakthrough [31], thereby attenuating the benefits of these drugs. In this case, RASi might confer their protective effect if hyperkalemia can be appropriately controlled and/or MRAs are used concomitantly, although such evidence is currently lacking. Further studies are warranted to elucidate the role of serum potassium levels on the efficacy of RASi.

A post-hoc analysis of the STOP-ACEi trial suggested the difference between ACEIs and ARBs [32]. While discontinuation of ACEIs showed an increased risk of kidney failure or KRT initiation, discontinuation of ARBs did not significantly affect kidney outcomes, though the statistical power was limited [32]. Since more than 80% of our patients received ARBs, our findings may not apply to discontinuation of ACEIs.

In contrast to the neutral association with kidney outcome, RASi discontinuation was significantly associated with higher mortality. Similar results have been reported in several observational studies although these studies did not specifically involve patients with advanced CKD, or their analytical methodologies were subject to bias unlike the target trial emulation approach [12–15, 17, 18]. Other observational studies reported higher mortality among those who discontinued RASi after eGFR decreased to <30 ml/min/1.73 m^2^ although the average serum potassium levels in these studies were within the normal range [33, 34]. The STOP-ACEi trial was underpowered to detect a difference in mortality [10]. Therefore, while further interventional studies are clearly needed, physicians should be cautious about discontinuing RASi since it may increase the risk of mortality.

We found a 17%-lower risk of severe hyperkalemia in the Discontinuation group. In other words, RASi continuation after developing hyperkalemia may enhance the risk of severe hyperkalemia even under the management by nephrologists who provided nutritional guidance, controlled metabolic acidosis, and used potassium binders appropriately to control hyperkalemia. These findings indicate that RASi-related hyperkalemia poses a clinical dilemma. While discontinuation may increase mortality risk, continuation may lead to severe hyperkalemia. Since mortality is a principal hard endpoint, RASi continuation might be encouraged. Notably, in patients under 70 years, a significant interaction was observed, with a higher risk of both kidney outcomes and mortality in those who discontinued RASi compared to those aged 70 years or older. RASi may provide less kidney-protective effects against atherosclerotic nephrocalcinosis, which is a common cause of CKD among older patients. Thus, RASi continuation may be less beneficial for older CKD patients. However, in our subgroup analysis, continuing RASi was associated with favorable outcomes even in patients with low urine protein, one of the features of atherosclerotic nephrocalcinosis, suggesting that age-related atherosclerosis does not fully explain the age-related differences. Older CKD patients are at a higher risk of AKI during RASi use than younger patients. This elevated AKI risk could offset the kidney-protective effect of continuing RASi. Non-cardiovascular deaths, such as deaths due to malignancies and infections, are prevalent in older patients, and these are unlikely to be reduced by RASi. It is possible that the association between RASi continuation and deaths was diluted by the predominance of non-cardiovascular deaths in the older subgroup. Therefore, in patients under 70, continuing RASi might be particularly important to consider, even in the presence of hyperkalemia, to mitigate the risks associated with discontinuation. Accordingly, as KDIGO has recently suggested [9], it might be reasonable to continue RASi while strengthening the preventive strategy for hyperkalemia to maximize the survival benefit of RASi. Novel potassium-binding agents, such as sodium zirconium cyclosilicate (SZC) and patiromer, are now available in Japan, making potassium management easier. SZC has been shown to improve the continuation rate of spironolactone therapy; however, given that an RCT indicated a possible increase in heart failure risk with SZC [35], its use should be approached with caution. For those who have difficulty controlling hyperkalemia by potassium-lowering therapy, however, RASi discontinuation should be considered.

The proportion of patients who discontinue RASi after developing hyperkalemia varies between studies, ranging from 13% to 35% [12–15, 17, 18]. Studies focusing on CKD populations tend to report lower discontinuation rates, whereas studies in the general population show higher discontinuation rates [12–15, 17, 18]. In our study, where all patients were managed by nephrologists, the discontinuation rate was 15%, which is consistent with previous studies in nephrology settings.

Our study has several limitations. First, despite the cloning, censoring, and weighting method including many baseline and time-dependent covariates, residual confounding cannot be ruled out. The significant association between RASi discontinuation and higher mortality might be confounded by a fact that older and sicker patients are more likely to discontinue the drug. However, after IPCW-weighting, almost all covariates including age were well-balanced between the 2 groups. Second, we could not confirm that the direct reason to discontinue RASi was hyperkalemia or other conditions like acute kidney injury or hypotension. Third, the causes of death were unknown. Fourth, RASi discontinuation was determined based on prescription data. Although prescription data could be a cause of misclassification, we have previously confirmed a high accuracy of these data through direct inspection [11]. Fifth, cases of pseudohyperkalemia could not be completely excluded. However, pseudohyperkalemia is less likely to occur in patients with impaired kidney function or those taking RASi [36]. Therefore, the number of pseudohyperkalemia cases in this study is considered to be limited. Finally, since our patients were cared by nephrology specialists in Japan, the generalizability of our findings to other countries clinical settings is uncertain.

### Perspective of Asia

The high prevalence of dialysis patients in Japan, South Korea, and Taiwan— placing them among the top three globally [37]— highlights the unique challenges in kidney disease management across Asia. Optimizing the use of renoprotective therapies, especially RASi, plays a key role in improving clinical outcomes in these regions.

In conclusion, discontinuing RASi after developing hyperkalemia was associated with a significantly higher risk of mortality despite a lower risk of severe hyperkalemia. It was not related to kidney outcome. Our findings provide insights into the optimal use of RASi for patients with hyperkalemia. Continuing RASi might be beneficial to improve survival if hyperkalemia is controllable, whereas discontinuation is a reasonable option for those with intractable hyperkalemia despite potassium-lowering therapy. Further studies are required to clarify the role of serum potassium levels on the efficacy of RASi.

## Supplementary information

Supplementary Information

## Data Availability

The Ethics Committee of Osaka University Hospital restricts public sharing of individual participant data because of privacy and ethical issues. The OCKR dataset and STATA program code used in this study are available on reasonable request to the corresponding author. Access to the data requires the approval of the OCKR study group.
