# Supplementary material for: Discontinuing renin-angiotensin system inhibitors after incident hyperkalemia and clinical outcomes: target trial emulation
Source: Hypertens Res. 2025 May 14;48(7):2034–44. doi: 10.1038/s41440-025-02218-8 (PMC12229888; doi:10.1038/s41440-025-02218-8)
Supplement: Supplementary file 1 — Supplementary Information [file 41440_2025_2218_MOESM1_ESM.docx]

**Supplemental Materials**

**Supplemental Tables**

Supplemental Table 1. Brief protocol of the target trial and its emulation using the OCKR

Supplemental Table 2. Demographic and clinical characteristics at the end of the grace period

Supplemental Table 3. Sensitivity analysis: Associations between RASi discontinuation after incident hyperkalemia and clinical outcomes after altering the grace period from 183 days to 100 days

Supplemental Table 4. Sensitivity analysis; Associations between RASi discontinuation after incident hyperkalemia and clinical outcomes in an intention-to-treat analysis

Supplemental Table 5. Sensitivity analysis; Associations between RASi discontinuation after incident hyperkalemia and clinical outcomes after excluding bicarbonate from the analysis

**Supplemental Figure**

Supplemental Figure 1. Target trial emulation with the cloning, censoring and weighting method

**Supplemental Table 1. Brief protocol of the target trial and its emulation using the OCKR**

| Component | **Target Trial (Randomized Controlled Trial)** | **Emulation using observational data** |
| --- | --- | --- |
| Eligibility | ***Inclusion criteria:***  Patients 1) aged 20 years or older, 2) who were prescribed RASi, 3) who developed hyperkalemia (serum potassium levels ≥5.5 mEq/L) for the first time since RASi were prescribed (index day), and 4) whose eGFR was ≥10 mL/min/1.73m² at the index day.  ***Exclusion criteria:***  Patients who had undergone KRT before the onset of hyperkalemia. | Patients registered in the OCKR database, who visited nephrology outpatient departments between January 1, 2005 and December 31, 2021. Otherwise, same as the target trial. |
| Treatment strategy | Discontinuing RASi within 183 days from the onset of hyperkalemia *versus* continuing RASi | In our main analysis, we compared a treatment strategy of discontinuing RASi within 183 days from the onset of hyperkalemia and remaining off during follow-up period *versus* that of continuing RASi during follow-up. |
| Treatment assignment | Eligible individuals are randomly assigned to one of the two strategies and are aware of the treatment strategy they are assigned to (i.e., no blinding). | Randomization was emulated by the cloning method. One replicate was assigned to the Discontinuation group and the other to the Continuation group. |
| Follow-up | Follow-up begins when patients are randomized (i.e., when hyperkalemia develops) and ends at the time of the outcome events or 5 years, whichever comes first. | Same as target trial. |
| Endpoints | ***Primary endpoint:***  A composite kidney outcome consisting of KRT initiation, a >50% decline in eGFR from the index day, or kidney failure (eGFR <5 mL/min/1.73m^2^).  ***Secondary endpoints****:*  All-cause death  severe hyperkalemia (serum potassium levels ≥6.5 mEq/L) | Same as target trial. |
| Causal contrasts | Intention-to-treat effect  Per-protocol effect | Same as target trial |
| Statistical analysis | Per-protocol analysis: Patients in the Discontinuation group are censored if RASi are not discontinued within the grace period and when RASi are restarted after discontinuation. Patients in the Continuation group are censored when RASi are discontinued. | Same as target trial.  Informative censoring was accounted for by inverse probability of censoring weights. |

Abbreviation: OCKR; Osaka Consortium for Kidney Disease Research; RASi, renin-angiotensin system inhibitors; KRT, kidney replacement therapy; eGFR, estimated glomerular filtration rate**Supplemental Table 2. Demographic and clinical characteristics at the end of the grace period**

|  | Before weighting | | |  | After weighting | | |
| --- | --- | --- | --- | --- | --- | --- | --- |
| Characteristics | Continuation group (n=1,521*) | Discontinuation group (n=310*) | SMD |  | Continuation group | Discontinuation group | SMD |
| Age (years) | 67 (13) | 72 (13) | 0.39 |  | 67 (13) | 66 (14) | 0.07 |
| Sex, male | 1,025 (67%) | 194 (63%) | 0.10 |  | 1,015 (67%) | 214 (69%) | 0.05 |
| Body mass index (kg/m^2^) | 22.9 (4.4) | 21.8 (4.2) | 0.24 |  | 22.7 (4.4) | 22.4 (3.8) | 0.06 |
| Diabetes mellitus | 547 (36%) | 89 (29%) | 0.16 |  | 533 (35%) | 122 (39%) | 0.07 |
| Systolic blood pressure (mmHg) | 137 (22) | 138 (22) | 0.06 |  | 137 (22) | 137 (22) | 0.005 |
| Diastolic blood pressure (mmHg) | 76 (14) | 75 (14) | 0.09 |  | 76 (14) | 77 (14) | 0.05 |
| ***Laboratory values*** |  |  |  |  |  |  |  |
| Hemoglobin (g/dL) | 11.4 (1.8) | 11.1 (1.6) | 0.15 |  | 11.4 (1.8) | 11.4 (1.8) | 0.04 |
| Sodium (mEq/L) | 140 (3) | 140 (3) | 0.02 |  | 140 (3) | 140 (3) | 0.10 |
| Potassium (mEq/L) | 4.9 (0.6) | 4.8 (0.6) | 0.18 |  | 4.9 (0.6) | 4.9 (0.6) | 0.05 |
| eGFR (mL/min/1.73m^2^) | 28 (16) | 30 (18) | 0.12 |  | 28 (16) | 28 (17) | 0.02 |
| Calcium (mg/dL) | 8.8 (0.7) | 8.8 (0.7) | 0.01 |  | 8.8 (0.7) | 8.8 (0.6) | 0.03 |
| Phosphorus (mg/dL) | 3.7 (0.8) | 3.7 (0.7) | 0.01 |  | 3.7 (0.7) | 3.8 (0.7) | 0.07 |
| Urate (mg/dL) | 6.9 (1.7) | 6.8 (1.9) | 0.05 |  | 6.9 (1.7) | 6.9 (1.9) | 0.02 |
| Bicarbonate (mEq/L) | 24.1 (3.7) | 24.3 (4.0) | 0.04 |  | 24.2 (3.8) | 24.0 (3.9) | 0.04 |
| UPCR (g/gCre) | 1.2 (0.4-2.9) | 0.9 (0.3-2.7) | 0.12 |  | 1.0 (0.3-2.5) | 0.9 (0.3-2.3) | 0.05 |
| ***Medications*** |  |  |  |  |  |  |  |
| MRAs | 216 (14%) | 36 (12%) | 0.08 |  | 213 (14%) | 31 (10%) | 0.09 |
| Calcium channel blockers | 996 (65%) | 120 (39%) | 0.56 |  | 917 (61%) | 180 (58%) | 0.1 |
| Beta blockers | 468 (31%) | 80 (26%) | 0.11 |  | 459 (30%) | 103 (33%) | 0.05 |
| Alfa blockers | 152 (10%) | 18 (6%) | 0.15 |  | 140 (9%) | 32 (10%) | 0.01 |
| SGLT2 inhibitors | 47 (2%) | 10 (3%) | 0.05 |  | 35 (2%) | 5 (2%) | 0.02 |
| Statins | 397 (26%) | 58 (19%) | 0.18 |  | 385 (25%) | 87 (28%) | 0.08 |
| Loop diuretics | 570 (37%) | 98 (32%) | 0.12 |  | 565 (37%) | 112 (38%) | 0.02 |
| Thiazide diuretics | 271 (18%) | 27 (9%) | 0.28 |  | 250 (16%) | 36 (11%) | 0.14 |
| Sodium bicarbonate | 346 (23%) | 50 (16%) | 0.17 |  | 332 (22%) | 84 (27%) | 0.05 |
| Potassium binding agents | 246 (16%) | 43 (14%) | 0.07 |  | 239 (16%) | 64 (21%) | 0.13 |
| Laxatives | 442 (29%) | 76 (25%) | 0.10 |  | 433 (29%) | 79 (26%) | 0.06 |
| NSAIDs | 2 (0%) | 1 (0%) | 0.04 |  | 2 (0%) | 0 (0%) | 0.004 |

Data are presented as n (%), mean (standard deviation), or median (IQR).

Abbreviations: SMD, standardized mean difference; eGFR, estimated glomerular filtration rate; UPCR, urinary protein-to-creatinine ratio; ACEIs, angiotensin-converting enzyme inhibitors; ARBs, angiotensin II receptor blockers; MRAs, mineralocorticoid receptor antagonists; SGLT2 inhibitors, sodium-glucose cotransporter 2 inhibitors; NSAIDs, non-steroidal anti-inflammatory drugs.

*Those who were censored or died within the grace period are not included in these data.

**Supplemental Table 3. Sensitivity analysis: Associations between RASi discontinuation after incident hyperkalemia and clinical outcomes after altering the grace period from 183 days to 100 days**

| Outcomes | Hazard ratio  (95% CI) | 5-year absolute risk, %  (95% CI) | 5-year risk difference, %  (95% CI) | 5-year risk ratio  (95% CI) |
| --- | --- | --- | --- | --- |
| **Primary outcome*** |  |  |  |  |
| Continuation group | Ref | 20.2 (15.7, 25.2) | Ref | Ref |
| Discontinuation group | 0.95 (0.71, 1.27) | 19.4 (14.9, 23.5) | -1.8 (-75.4, 4.4) | 0.92 (0.74, 1.26) |
| **KRT** |  |  |  |  |
| Continuation group | Ref | 10.5 (8.4, 14.0) | Ref | Ref |
| Discontinuation group | 1.14 (0.81, 1.60) | 12.3 (9.0, 15.5) | 1.2 (-2.3, 5.0) | 1.11 (0.83, 1.58) |
| **eGFR** ≥ **50% decline** |  |  |  |  |
| Continuation group | Ref | 15.1 (11.8, 18.5) | Ref | Ref |
| Discontinuation group | 0.93 (0.66, 1.31) | 13.3 (10.2, 17.7) | -2.0 (-6.0, 2.3) | 0.88 (0.68, 1.16) |
| **eGFR < 5 mL/min/1.73m^2^** |  |  |  |  |
| Continuation group | Ref | 5.6 (3.3, 9.0) | Ref | Ref |
| Discontinuation group | 1.13 (0.65, 1.95) | 5.8 (3.1, 9.5) | 0.1 (-3.0, 3.3) | 1.02 (0.55, 1.80) |
| **All-cause death** |  |  |  |  |
| Continuation group | Ref | 38.9 (33.2, 44.2) | Ref | Ref |
| Discontinuation group | 1.16 (1.00, 1.36) | 46.3 (39.8, 50.6) | 7.1 (0.7, 13.1) | 1.18 (1.01, 1.38) |
| **Severe hyperkalemia**** |  |  |  |  |
| Continuation group | Ref | 28.8 (22.4, 36.) | Ref | Ref |
| Discontinuation group | 0.88 (0.72, 1.11) | 24.4 (18.0, 31.0) | -4.1 (-13.0, 3.0) | 0.84 (0.62, 1.10) |

*A composite kidney outcome consisting of KRT initiation, a >50% decline in eGFR from the index day, or kidney failure (eGFR <5mL/min/1.73m^2^).

** Severe hyperkalemia was defined as serum potassium levels of >6.5 mEq/L

Abbreviations: CI, confidence interval; KRT, kidney replacement therapy; eGFR, estimated glomerular filtration rate; RASi; renin-angiotensin system inhibitors.

**Supplemental Table 4. Sensitivity analysis; Associations between RASi discontinuation after incident hyperkalemia and clinical outcomes in an intention-to-treat analysis**

| Outcomes | Hazard ratio  (95% CI) | 5-year absolute risk, %  (95% CI) | 5-year risk difference, %  (95% CI) | 5-year risk ratio  (95% CI) |
| --- | --- | --- | --- | --- |
| **Primary outcome*** |  |  |  |  |
| Continuation group | Ref | 23.8 (19.3, 28.7) | Ref | Ref |
| Discontinuation group | 0.93 (0.76, 1.14) | 22.0 (18.5, 26.2) | -1.7 (-5.4, 2.4) | 0.93 (0.79, 1.12) |
| **KRT** |  |  |  |  |
| Continuation group | Ref | 13.4 (10.6, 16.3) | Ref | Ref |
| Discontinuation group | 0.96 (0.73, 1.24) | 12.9 (10.1, 16.2) | -0.5 (-3.5, 2.5) | 0.93 (0.75, 1.21) |
| **eGFR** ≥ **50% decline** |  |  |  |  |
| Continuation group | Ref | 18.6 (14.8, 21.8) | Ref | Ref |
| Discontinuation group | 0.95 (0.76, 1.18) | 17.2 (13.7, 21.2) | -1.3 (-4.6, 2.6) | 0.93 (0.79, 1.15) |
| **eGFR < 5 mL/min/1.73m^2^** |  |  |  |  |
| Continuation group | Ref | 8.3 (5.9, 11.4) | Ref | Ref |
| Discontinuation group | 0.81 (0.57, 1.15) | 6.7 (4.0, 9.0) | -1.7 (-4.0, 0.5) | 0.79 (0.56, 1.09) |
| **All-cause death** |  |  |  |  |
| Continuation group | Ref | 38.5 (33.2, 45.4) | Ref | Ref |
| Discontinuation group | 1.14 (1.00, 1.31) | 46.0 (40.0, 53.4) | 8.0 (0.5, 13.0) | 1.20 (1.01, 1.36) |
| **Severe hyperkalemia**** |  |  |  |  |
| Continuation group | Ref | 30.4 (24.9, 36.8) | Ref | Ref |
| Discontinuation group | 0.87 (0.71, 1.05) | 24.7 (17.7, 30.0) | -6.2 (-15.0, -0.6) | 0.79 (0.56, 0.98) |

*A composite kidney outcome consisting of KRT initiation, a >50% decline in eGFR from the index day, or kidney failure (eGFR <5mL/min/1.73m^2^).

** Severe hyperkalemia was defined as serum potassium levels of >6.5 mEq/L

Abbreviations: CI, confidence interval; KRT, kidney replacement therapy; eGFR, estimated glomerular filtration rate; RASi; renin-angiotensin system inhibitors.

**Supplemental Table 5. Sensitivity analysis; Associations between RASi discontinuation after incident hyperkalemia and clinical outcomes after excluding bicarbonate from the analysis**

| Outcomes | Hazard ratio  (95% CI) | 5-year absolute risk, %  (95% CI) | 5-year risk difference, %  (95% CI) | 5-year risk ratio  (95% CI) |
| --- | --- | --- | --- | --- |
| **Primary outcome*** |  |  |  |  |
| Continuation group | Ref | 17.8 (14.5, 21.6) | Ref | Ref |
| Discontinuation group | 0.99 (0.81, 1.22) | 18.0 (15.2, 21.1) | 0.2 (-3.8, 3.7) | 1.02 (0.80, 1.21) |
| **KRT** |  |  |  |  |
| Continuation group | Ref | 10.1 (8.2, 12.3) | Ref | Ref |
| Discontinuation group | 0.94 (0.71, 1.23) | 9.6 (7.2, 12.8) | -0.4 (-2.6, 2.0) | 0.95 (0.76, 1.19) |
| **eGFR** ≥ **50% decline** |  |  |  |  |
| Continuation group | Ref | 13.9 (11.5, 16.6) | Ref | Ref |
| Discontinuation group | 1.03 (0.82, 1.29) | 14.1 (11.8, 16.4) | 0.2 (-2.7, 2.5) | 1.02 (0.82, 1.20) |
| **eGFR < 5 mL/min/1.73m^2^** |  |  |  |  |
| Continuation group | Ref | 5.5 (3.9, 8.3) | Ref | Ref |
| Discontinuation group | 0.94 (0.63, 1.39) | 5.1 (3.5, 7.0) | -0.4 (-2.8, 1.9) | 0.90 (0.62, 1.42) |
| **All-cause death** |  |  |  |  |
| Continuation group | Ref | 39.0 (34.4, 43.3) | Ref | Ref |
| Discontinuation group | 1.16 (1.00, 1.33) | 45.7 (41.2, 49.8) | 6.0 (2.2, 11.7) | 1.15 (1.05, 1.32) |
| **Severe hyperkalemia**** |  |  |  |  |
| Continuation group | Ref | 29.6 (22.9, 34.1) | Ref | Ref |
| Discontinuation group | 0.84 (0.68, 0.99) | 23.5 (17.6, 27.1) | -6.1 (-10.7, 0.5) | 0.79 (0.65, 0.98) |

*A composite kidney outcome consisting of KRT initiation, a >50% decline in eGFR from the index day, or kidney failure (eGFR <5mL/min/1.73m^2^).

** Severe hyperkalemia was defined as serum potassium levels of >6.5 mEq/L

Abbreviations: CI, confidence interval; KRT, kidney replacement therapy; eGFR, estimated glomerular filtration rate; RASi; renin-angiotensin system inhibitors.

**Supplemental Figure 1. Target trial emulation with the cloning, censoring and weighting method**


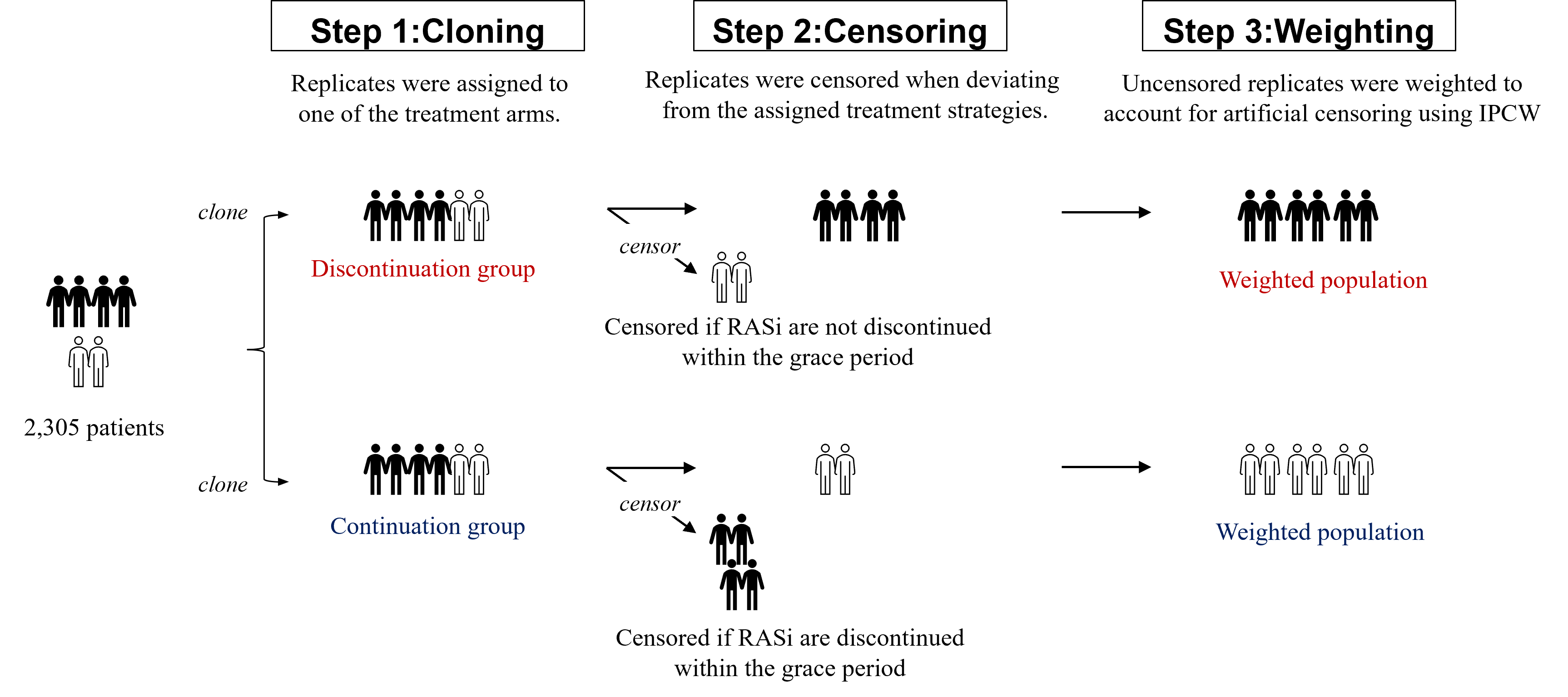


To emulate the target trial comparing the Discontinuation and Continuation group using observational data, we used the cloning, censoring, and weighting method. Step 1, Cloning: Each eligible individual was cloned into two identical replicates on the index day when hyperkalemia developed. One replicate was assigned to the Discontinue group and another to the Continuation group. This step emulates randomization in clinical trials and addresses immortal time bias. Step 2, Censoring: Replicates were censored if they deviated from the assigned treatment strategy during the grace period. This artificial censoring would lead to selection bias. Step 3, Weighting: Selection bias was dealt with inverse probability of censoring weights (IPCW), which are the reciprocal of the probability of being uncensored, estimated from a pooled logistic regression. This model included baseline and time-dependent covariates as well as time (linear and quadratic terms). The IPCW were stabilized by multiplying them by the probability of being uncensored based on pooled logistic regression models including baseline covariates only. The IPCW at each month were calculated by multiplying all IPCW from the index day up to a specific month. These weights created a pseudo-population similar to the full uncensored population to remove the selection bias. To avoid undue influence of outliers, IPCW were truncated at the 99th percentile.

Abbreviations: RASi, renin angiotensin system inhibitors; IPCW, inverse probability censoring weights.
